# Role of glycolysis in inflammatory bowel disease and its associated colorectal cancer

**DOI:** 10.3389/fendo.2023.1242991

**Published:** 2023-10-10

**Authors:** Yuxuan Xia, Li Zhang, Dickson Kofi Wiredu Ocansey, Qiang Tu, Fei Mao, Xiumei Sheng

**Affiliations:** ^1^ Key Laboratory of Medical Science and Laboratory Medicine of Jiangsu Province, School of Medicine, Jiangsu University, Zhenjiang, Jiangsu, China; ^2^ Nanjing Lishui People’s Hospital, Zhongda Hospital Lishui Branch, Southeast University, Nanjing, Jiangsu, China; ^3^ Directorate of University Health Services, University of Cape Coast, Cape Coast, Ghana; ^4^ Clinical Laboratory, Nanjing Jiangning Hospital, Nanjing, Jiangsu, China

**Keywords:** glycolysis, colorectal cancer, inflammatory bowel disease, immunity, intestinal

## Abstract

Inflammatory bowel disease (IBD) has been referred to as the “green cancer,” and its progression to colorectal cancer (CRC) poses a significant challenge for the medical community. A common factor in their development is glycolysis, a crucial metabolic mechanism of living organisms, which is also involved in other diseases. In IBD, glycolysis affects gastrointestinal components such as the intestinal microbiota, mucosal barrier function, and the immune system, including macrophages, dendritic cells, T cells, and neutrophils, while in CRC, it is linked to various pathways, such as phosphatidylinositol-3-kinase (PI3K)/AKT, AMP-activated protein kinase (AMPK), mammalian target of rapamycin (mTOR), and transcription factors such as p53, Hypoxia-inducible factor (HIF), and c-Myc. Thus, a comprehensive study of glycolysis is essential for a better understanding of the pathogenesis and therapeutic targets of both IBD and CRC. This paper reviews the role of glycolysis in diseases, particularly IBD and CRC, via its effects on the intestinal microbiota, immunity, barrier integrity, signaling pathways, transcription factors and some therapeutic strategies targeting glycolytic enzymes.

## 1 Introduction

There has been increasing interest in studying the differences between tumor tissue metabolism and normal tissue metabolism, also known as metabolic reprogramming (1) This reprogramming mainly involves lipid metabolism (2), amino acid metabolism (3), and glycolysis (4). Among them, glycolysis is closely related to the energy metabolism of tumor cells, as first discovered by Professor Otto H. Warburg in the 1920s (5). He found that the metabolism of tumor cells differs from that of normal cells, which generally only undergo glycolysis for energy production under hypoxia. In a normal oxygen environment, the more efficient oxidative phosphorylation pathway (OXPHOS) provides energy. Interestingly, tumor cells with high energy demand still choose glycolysis as the main energy supply pathway even under hypoxic conditions, a phenomenon known as the Warburg effect (6). Nearly a century has passed since this discovery, and as research continues, the Warburg effect is more accurately understood. Contrary to the initial view of Warburg’s paper, the enhanced glycolysis in tumor cells is not a result of compensation for differential energy production by abnormally functioning mitochondria. Instead, it is due to the high expression of HIF-1, the activation of oncogenes, the mutation of oncogenes, and the action of related signaling pathways (7). Studying the mechanisms behind it could clarify the role of glycolysis in related diseases.

Inflammatory bowel diseases (IBD) are chronic, non-specific inflammatory diseases of the intestinal system that are still being explored for better understanding in terms of their etiology and pathogenesis. The two main types of IBD are ulcerative colitis (UC) and Crohn’s disease (CD) (8). IBD is a global disease with varying epidemiological characteristics in different regions. Since 2020, IBD has been in the ‘Acceleration in Incidence stage’ in most newly industrialized countries in Asia and Latin America, with the incidence increasing more rapidly (9). IBD is characterized as an indolent and recurrent disease and radical drug therapy has not yet been established, leaving some patients in a state of lifelong disease. Furthermore, patients with IBD have a higher risk of developing colorectal cancer (CRC), also known as colitis-associated colorectal cancer (CAC) (10, 11). According to the most recent data published by the American Cancer Society in 2022, CRC is one of the three most prevalent cancers and has one of the largest racial disparities in treatment outcomes (12). Patients with CRC have a high mortality rate and are the third most common cause of cancer deaths worldwide, with nearly one million deaths per year (13). The application of current therapies in the clinical treatment of IBD does not completely relieve the symptoms of the disease (14), and new treatments are being researched and validated (15).

The regulation of the glycolytic pathway, an important mechanism in both intestinal inflammation and tumors, is one of the therapeutic targets in IBD and CRC (16). We review the general role of glycolysis in diseases, focusing on its effects on key gastrointestinal components such as microbiota, immunity, barrier integrity, and signal molecules. Further investigation of the molecular mechanisms involved in these regulations could help explore and discover effective therapeutic targets for IBD and CRC.

## 2 Glycolysis

### 2.1 Process and significance

**Figure 1:**
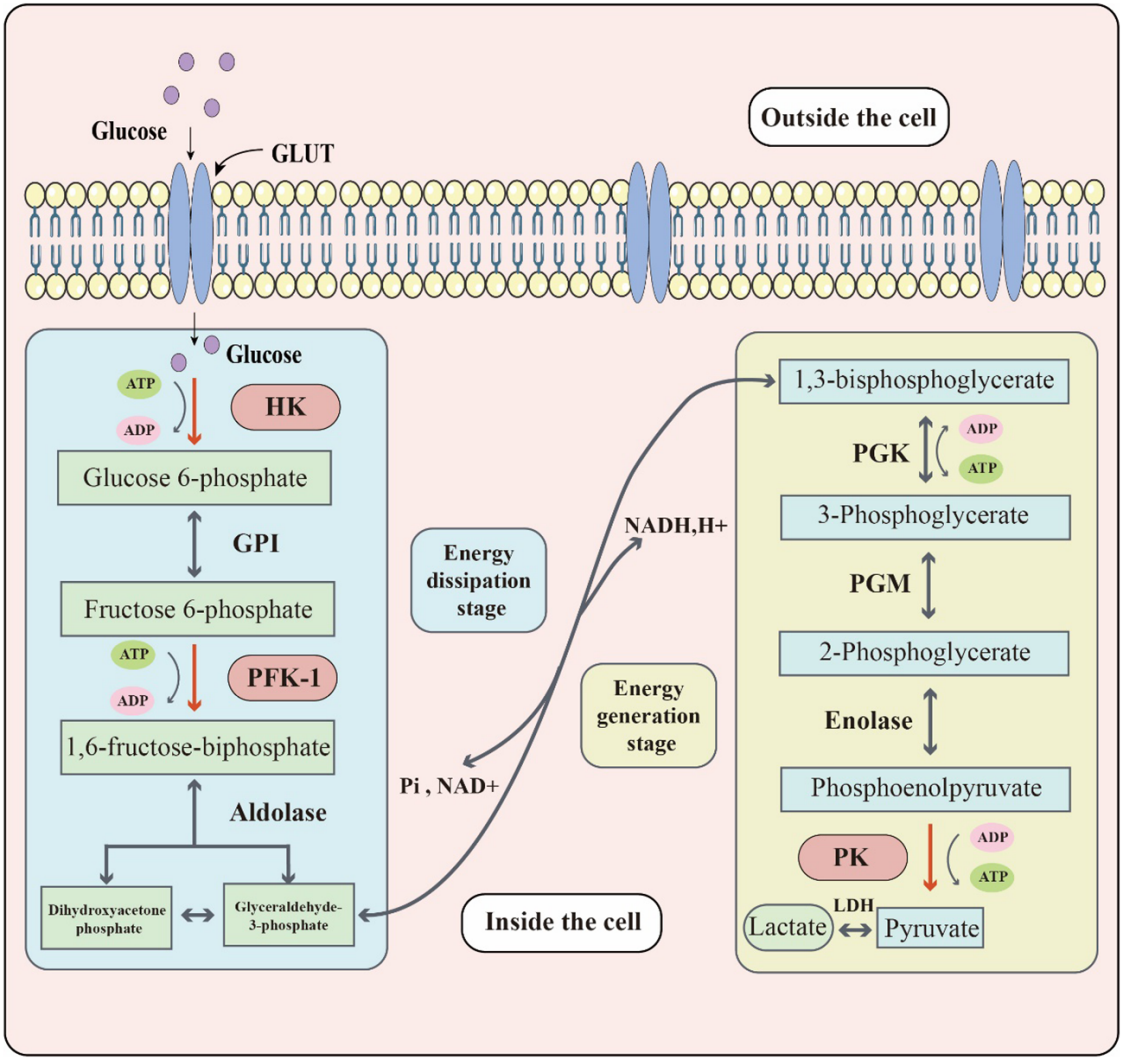
The key biochemical process of glycolysis. Glucose located outside the cell enters the cell through the glucose transporter (GLUT). Glucose reacts with glycolytic enzymes such as hexokinase (HK), phosphofructokinase-1 (PFK-1) & pyruvate kinase (PK) to provide energy to the cell. The whole glycolytic process can be divided into the energy dissipation stage and the energy generation stage.

Sugar metabolism in organisms involves several processes, such as glycolysis, aerobic oxidation, pentose phosphate pathway, and glycogen synthesis and catabolism. Glycolysis, in particular, is the process of breaking down glucose or glycogen to produce lactate and energy under relatively anoxic conditions. This process can be divided into two stages: the first stage converts glucose or glycogen into pyruvate through the action of various enzymes, and the second stage reduces pyruvate to lactate via lactate dehydrogenase (LDH) activity. Through this process, one molecule of glucose can produce two molecules of ATP (17). Three key enzymes namely, hexokinase (HK), phosphofructokinase-1 (PFK1), and pyruvate kinase (PK) determine the rate of reaction in the specific biochemical process: (**Figure 1**). Although the entire reaction process is mostly reversible, the three key enzymes catalyze irreversible reactions. Glucose transporters are also crucial for glycolysis since they are responsible for transporting glucose, the raw material, across membranes. Around 14 different glucose transporters have been identified, among which GLUT1 is widely expressed in human tissues and organs and serves as the primary glucose transporter (18). Although glycolysis produces less energy compared to the aerobic oxidation of sugar, it plays an important role in living organisms and has unique and significant physiological significance. In times of relative hypoxia, such as during strenuous exercise like rock climbing, where the oxygen supply is relatively inadequate, muscle tissue heavily relies on glycolytic processes for energy supply (19). Some cells or tissues, such as mature red blood cells, retina, and renal medulla, also rely heavily on glycolytic processes for energy supply under aerobic conditions.

### 2.2 Glycolysis-associated diseases

Glycolysis has an important impact on the physiological functions and disease processes of living organisms. The glycolytic process provides the energy required by organisms and is the basis for their survival and activity. However, in some diseases, abnormalities in the glycolytic process may lead to the disruption of physiological functions and adversely affect the organism. Changes in glycolysis in some representative diseases are shown in **Table 1** below.

**Table 1:** Changes and effects of glycolysis in various diseases.

Disease classification	Disease Name	Glycolysis variation trend	Key effects	References
Metabolic diseases	Type 2 diabetes	**↑**	Reduces insulin release; Impairs β-cell metabolism	(20)
Neurological diseases	Alzheimer’s Disease	**↓**	Impairs l-serine biosynthesis pathway; Synapse loss	(21)
	Parkinson’s disease	**↓**	Decreases brain ATP levels; Neuron loss	(22)
Vascular disease	Atherosclerosis	**↑**	Promotes endothelial cell proliferation; Protects endothelial barrier integrity	(23)
	Calcific aortic valve disease	**↑**	Promotes valve calcification	(24)
	Ocular neovascular diseases	**↑**	Promotes ocular neovascularization	(25)
Neoplastic Disease	Gastric cancer	**↑**	Promotes growth and migration	(26)
	Pancreatic cancer	**↑**	Promotes growth and migration	(27)
	Breast cancer	**↑**	Promotes growth and migration	(28)
	Colorectal cancer	**↑**	Promotes growth and migration	(29)
	Prostatic cancer	**↑**	Promotes growth and migration	(30)
Digestive Diseases	Inflammatory bowel disease	**↑**	Proinflammatory	(31)
	Colorectal cancer	**↑**	Promotes growth and migration; Induced resistance	(32) (33)

#### 2.2.1 Metabolic diseases

As a metabolic process, glycolysis is of great importance in metabolic diseases. A typical example of a metabolic disorder is Diabetes mellitus, which is characterized by hyperglycemia caused by multiple factors. Diabetes can be divided into two main types, type 1 and type 2 diabetes, and 90% of patients are diagnosed with type 2 diabetes (34). Type 2 diabetes is a serious public health problem. Although a decrease in pancreatic β-cells is observed in type 2 diabetes, it is not sufficient to explain the decrease in insulin secretion, which is mainly due to altered pancreatic β-cell metabolism and impaired cell function (35, 36). Studies have shown (20) that glycolysis is one of the most significantly upregulated metabolic pathways in type 2 diabetic islets, and many glycolysis-related enzymes show significant elevations at both protein and mRNA levels. These results are also observed in a Goto-Kakizaki (GK) rat model (37). Some glycolysis-related metabolites, such as hexose monophosphate, 3-phosphoglycerate, and lactate, have been found to have a high correlation with the risk of type 2 diabetes (38).

#### 2.2.2 Neurological diseases

Glycolysis is also implicated in neurological disorders. Alzheimer’s disease (AD) is a progressive neurological disorder characterized by memory loss and cognitive dysfunction. In the early stages of AD, reduced glycolysis is commonly observed, and impaired glycolytic metabolism due to reduced glycolytic flux may be inherent to the pathogenesis of AD (21). Glial cells, which are closely associated with AD (39), utilize the glycolytic intermediate 3-phosphoglycerate to produce the amino acid l-serine. Reduced glycolysis leads to a corresponding decrease in l-serine synthesis, as demonstrated in mouse models of AD and AD patients (21, 40). Additionally, glycolysis plays a critical role in the treatment of Parkinson’s disease, and terazosin has been shown to activate the enzymatic activity of phosphoglycerate kinase 1 (PGK1) (22, 41). This activation increases the production of pyruvate, a glycolytic product, which further stimulates oxidative phosphorylation, mitochondrial activity, and ATP production. These changes may have a direct impact on the pathophysiology of PD.

#### 2.2.3 Vascular diseases

Glycolysis is also relevant in the study of various vascular diseases. Atherosclerosis (AS) is a common vascular disease, and among many causative factors, endothelial cell injury in arteries is considered a major trigger for its development (42). Endothelial cell proliferation relies mainly on glycolysis for energy supply to meet the energy requirements for growth. Reducing glycolysis by knocking down pharmacological activation of the protein kinase AMP-activated α1 (PRKAA1) in endothelial cells blocks endothelial cell proliferation and accelerates atherosclerotic lesion formation in hyperlipidemic mice. Conversely, upregulating solute carrier family 1 member 2 (SLC1A2) expression enhances impaired glycolysis in PRKAA1-deficient endothelial cells, leading to enhanced endothelial cell viability, endothelial cell barrier integrity, and reversal of atherosclerotic susceptibility (23). Glycolytic metabolism also provides important directions in drug development and mechanistic studies of calcific aortic valve disease (24), and ocular neovascular disease (25).

#### 2.2.4 Neoplastic diseases

Glycolysis is a major focus of research in various tumors and a popular subject of study. The effect of glycolysis on tumors is complex. On one hand, the metabolism of tumor cells is characterized by a high growth rate and high metabolism, requiring a large amount of energy, making glycolysis an important process in tumors. On the other hand, the glycolytic pathway of tumor cells is usually abnormal, leading to an increased demand for sugar in tumor cells, while the metabolism of normal cells is suppressed (43, 44). Wang et al. (26) found that forkheadbox O4 (FOXO4) could inhibit the rate of glycolysis in gastric cancer cells by directly inhibiting the glycolytic enzyme lactate dehydrogenase (LDH) A and identified the HIF-1α-FOXO4-LDHA axis. Highly active glycolysis in pancreatic cancer produces a large number of metabolites and drives tumor cell invasion and migration. Important enzymes and intermediates in the glycolytic process can affect the metastasis of pancreatic cancer by participating in signal transduction or epigenetic regulation related to epithelial-mesenchymal transition (EMT), angiogenesis, and colonization (27, 45). In studying breast cancer-related mechanisms, Jiang et al. (28) found that Zeb1 mobilizes glycolytic activity through the PI3K/Akt/HIF-1α signaling axis, driving the formation of an immunosuppressive tumor microenvironment (TME). The expression of Zeb1 was positively associated with glycolytic dysregulation and the accumulation of M2-like tumor-associated macrophages (TAMs). Moreover, glycolysis is being intensively investigated in numerous other cancers, such as CRC (29) and prostate cancer (30).

#### 2.2.5 Digestive diseases

Digestive system diseases are a major threat to human health. According to relevant epidemiological statistics, digestive system diseases exist in more than one-third of epidemic cases (46). The relatively high incidence means that digestive diseases represent a significant public health burden. Therefore, the related research on digestive system diseases is of great significance. IBD and CRC are serious diseases closely related to the digestive system, and their causes are complex and diverse, including metabolic factors. Glycolysis, a key metabolic pathway, has been found to play an important regulatory role in both diseases. In subsequent parts of this paper, the regulation mechanism of glycolysis in IBD and CRC is expounded to provide more inspiration for the research and treatment of these diseases.

## 3 Role of glycolysis in IBD

**Figure 2:**
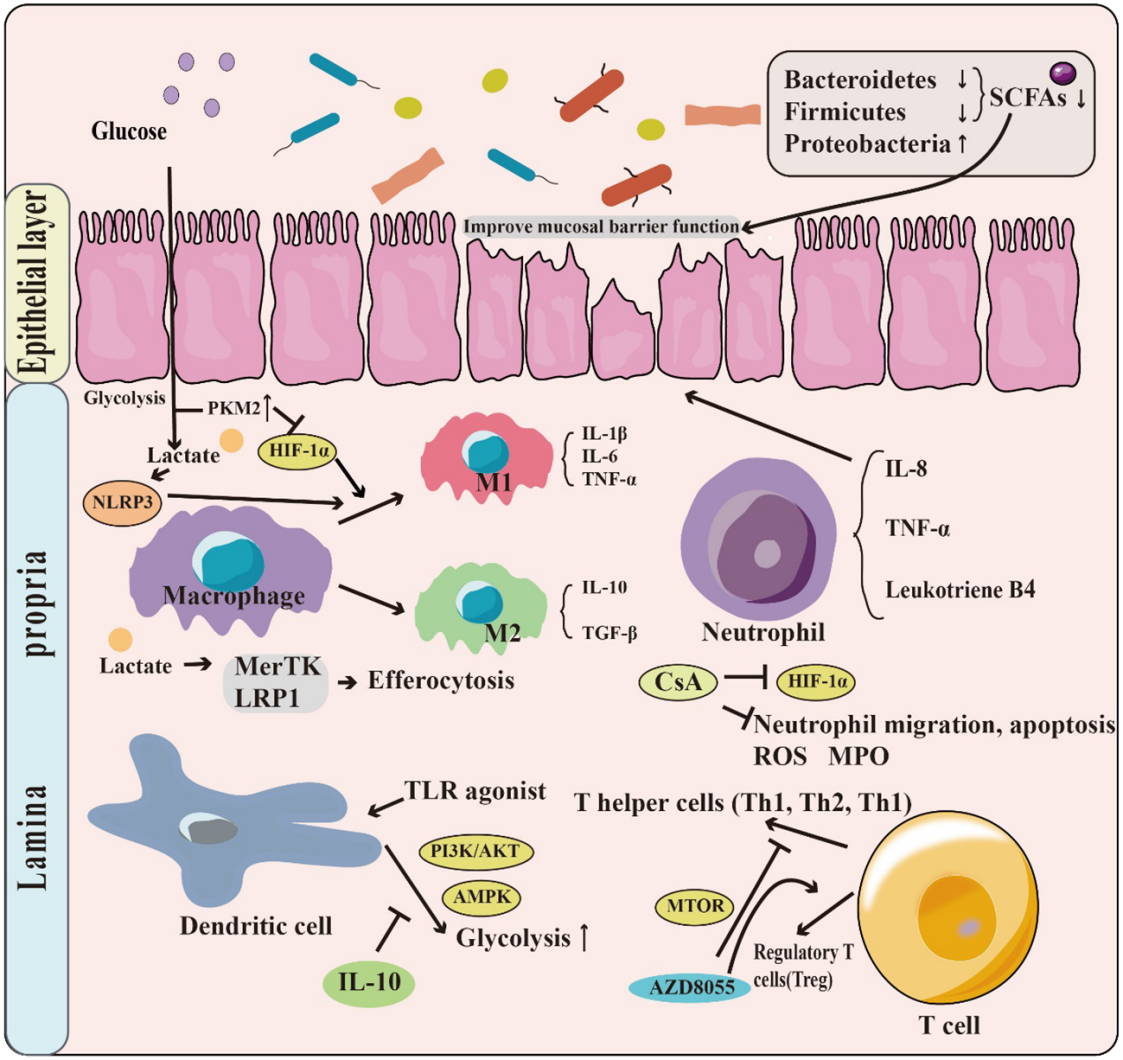
The role of glycolysis in IBD. Patients with IBD have a change in the structure and abundance of the intestinal microbiota, with phylum-level decreases in the Firmicutes & Bacteroides but increases in Proteobacteria. This leads to a decrease in the production of short-chain fatty acids (SCFAs), which have the function of promoting the intestinal barrier. Moreover, glycolysis has a crucial role in regulating immune system functions. For example, T Lactate, a product of glycolysis, can contribute to the M1 phenotype polarization by promoting NLRP3 activation. Glycolysis is also involved in the regulation of neutrophil, dendritic cell, and T cell populations.

Glycolysis plays a vital role in supporting the growth and metabolism of intestinal microbiota by producing energy and metabolites. However, the composition of the intestinal microbiota in patients with IBD differs in structure from that of healthy individuals, which can negatively affect the balance of intestinal homeostasis. For instance, alteration in the intestinal microbiota impacts the synthesis of short-chain fatty acids (SCFAs), which play an essential role in maintaining intestinal health. Moreover, glycolysis has a crucial role in regulating immune system functions. The glycolytic metabolic pathway can regulate the polarization of macrophages, which affects their activation status and cellular function. Glycolysis is also involved in the regulation of neutrophil, dendritic cell, and T cell populations and their cellular functions. Additionally, glycolysis has a unique mechanism for intestinal barrier function (**Figure 2**).

### 3.1 Effects of glycolysis on intestinal microbiota

IBD is a chronic inflammatory disease with its pathogenesis not fully understood. The human intestinal microbiota is a complex ecosystem that plays a crucial role in host physiology, including the regulation of immune responses. Dysbiosis of the intestinal microbiota, characterized by changes in microbial composition and diversity, is usually associated with the development of IBD (47). Targeted treatment of the intestinal microbiota may be an effective way to treat IBD (48). The species diversity of intestinal microbiota in patients with IBD is lower than that in healthy people. There are phylum-level decreases in Firmicutes (*Faecalibacterium*), Bacteroides (*Bacteroides*), and Fusobacteria, and increases in Proteobacteria (adherent invasive *E. coli)*, Actinobacteria, and Verrucomicrobia (49–51). Members of the Firmicutes produce short-chain fatty acids (SCFAs), important metabolites for maintaining intestinal homeostasis. SCFAs are the main bacterial metabolites produced by specific colonic anaerobic bacteria after fermentation of dietary fiber and resistant starch and mainly include acetate, propionate, and butyrate. Members of the Bacteroidetes mainly produce acetate and propionate and members of the Firmicutes mainly produce butyrate in the human intestine (52–54). The role of SCFAs in the prevention and treatment of IBD is increasingly recognized (55), and their inhibitory effect on the growth of *Salmonella typhi*, pathogenic *Escherichia coli*, and *Clostridium difficile* has been demonstrated (56). Regulation of SCFA production and transport is essential for maintaining the balance of the intestinal microbial community and host immunity. The process of SCFA production involves the metabolism of dietary fiber by the intestinal microbiota and several enzymes and transporter proteins, with glycolysis playing an important role.

Glycolysis is the process of catabolizing glucose into pyruvate, which can be further metabolized to produce SCFAs. Pyruvate is converted to acetyl coenzyme A, which serves as a feedstock substrate for the biosynthesis of acetate, propionate, and butyrate. The activity of glycolysis can be regulated by various factors, including the expression of genes encoding enzymes involved in the process. Pyruvate kinase M2 (PKM2) is a crucial glycolysis-related enzyme and mediator of the inflammatory process. Serum PKM2 levels are six-fold higher in patients with IBD than in healthy population controls (57). PKM2 upregulation increases glycolytic activity and provides more raw material for the synthesis of SCFAs. However, the production of SCFAs is usually lower in IBD patients than in healthy populations due to changes in the composition of the intestinal microbiota and reduced dietary fiber intake. Modulation of glycolysis and SCFA production is a promising avenue for the development of new treatments for IBD (58). Biological agents such as prebiotics can be considered to promote the growth of SCFAs-producing bacteria in the gut, thereby alleviating IBD symptoms.

### 3.2 Effects of glycolysis on the immune system

The glycolytic pathway has an important impact on the activation and regulation of the immune system, especially in the context of IBD. As a metabolic process, glycolysis has the potential to regulate the immune system and changes in glycolysis can cause alterations in the immune system response. This may lead to the development of IBD, which is characterized by chronic inflammation of the intestine. Glycolysis affects important immune cells, such as macrophages, neutrophils, dendritic cells, and regulatory T cells.

#### 3.2.1 Macrophages

Macrophages are an important class of immune cells, and their role in IBD is significant. They are distributed in the submucosal tissues of the intestine and play a key role in the immune response of the intestinal mucosa (59). In the intestinal inflammatory response, macrophages can enhance the inflammatory response by inducing the release of multiple cytokines and inflammatory mediators. In the M1 polarized state, macrophages produce various pro-inflammatory cytokines such as IL-1β, IL-6, and TNF-α (60), which exacerbate the inflammatory response. In contrast, in the M2 polarized state, macrophages secrete anti-inflammatory cytokines such as IL-10 and TGF-β (61, 62), promoting tissue repair. During IBD, an imbalance of oxygen supply and demand in the mucosal microenvironment leads to severe hypoxia at the inflamed site (63). In a hypoxic environment, macrophages undergo metabolic reprogramming to meet energy demands, especially in M1 macrophages, their focus shifts from oxidative phosphorylation metabolism to aerobic glycolysis (64).

Glycolysis plays an important role in macrophage polarization. The glycolytic product, lactate, affects macrophage polarization, and PYD domains-containing protein 3 (NLRP3) inflammasome mediates macrophage polarization in innate immunity. NLRP3 activation requires glycolytic downstream metabolism, including lactate fermentation and pyruvate oxidation (65). In one study, it was found that an imbalance in M1/M2 macrophage polarization caused by increased M1- and decreased M2-type macrophage, is involved in the development of IBD (66). In LPS and IFN-γ stimulated bone marrow-derived macrophage (BMDM) cell models of inflammation, mRNA expression of glycolytic enzyme genes, including glucose transporter protein 1 (*GLUT1*), enolase 1 (*ENO1)*, pyruvate kinase (*PKM*), pyruvate dehydrogenase kinase 1 (*PDK1*), aldolase, lactate dehydrogenase A (*LDHA*), phosphoglycerate mutase (*PGAM*), phosphofructokinase (*PFK*), and glyceraldehyde-3-phosphate dehydrogenase (*GhAPDH*) were significantly elevated, indicating that glycolysis in macrophages is significantly elevated in the IBD state. Further studies revealed that the HIF-1α/glycolytic axis was responsible for inhibiting macrophage M1 polarization and thus repair of IBD (31). Among the glycolytic enzymes, the value of PKM2 in IBD treatment has been emphasized. HIF-1α-mediated glycolytic reprogramming can be regulated by PKM2, forming PKM2-HIF-1α complexes in the nucleus (67). Meanwhile, PKM2 desuccinylation via SIRT5 inhibits macrophage IL-1β production and can prevent the development of DSS-induced colitis (68). In addition to PKM2, recent studies have found that macrophage efferocytosis promotes glycolysis that is dependent on the rapid activation of phosphofructose-2-kinase/fructose-2,6-bisphosphatase 2 (PFKFB2), which is distinct from glycolysis in pro-inflammatory macrophages. The bone marrow of mice with activation-deficient PFKFB2 exhibits impaired efferocytosis, suggesting that PFKFB2-mediated glycolysis is associated with efferocytosis. *In vitro* experiments have shown that glycolysis in apoptotic cells promotes sustained efferocytosis through lactate-mediated upregulation of macrophage c-mer tyrosine kinase (MerTK) and lipoprotein receptor-related protein 1 (LRP1). Therefore, macrophage efferocytosis-induced glycolysis is a unique metabolic process and a promising therapeutic idea for IBD (69).

#### 3.2.2 Neutrophils

White blood cells in the blood are neutrophils, possessing great importance. However, in inflammatory diseases, neutrophils are a double-edged sword, properly wielded as a powerful weapon to protect the body, but when they are not strictly regulated, they become a destructive force. In IBD, the accumulation of neutrophils in epithelial crypts and the intestinal lumen is directly related to epithelial damage, so neutrophil migration across the mucosal epithelium can be a hallmark of inflammatory diseases such as UC and CD (70, 71). At the same time, neutrophils produce high levels of reactive oxygen species (ROS), many proteases, and pro-inflammatory cytokines and mediators (e.g., IL-8, TNF-α, and leukotriene B4), which can lead to the disruption of the epithelial barrier (72). However, as a double-edged sword, there is also a good side to neutrophils, and one study reported (73) that patients with IBD had more CD177+ neutrophils in peripheral blood and inflamed mucosa compared to healthy controls. CD177+ neutrophils are a functionally activated population that can play a protective role in IBD by increasing bactericidal activity, such as ROS, antimicrobial peptides, and neutrophil extracellular traps. Additionally, IL-22 production in IBD can play a protective role, and targeting CD177+ neutrophils may be a potential therapeutic strategy for IBD.

Neutrophils are traditionally considered short-lived cells with high protein synthesis activity, and polymorphonuclear neutrophils (PMN) have a low number of mitochondria. Most of their required ATP is produced by glycolysis (74). Therefore, the glycolytic pathway can be used to influence neutrophil function and achieve the goal of repairing IBD. Recently, it was found that cyclosporine A (CsA) upregulates HIF-1α expression and glycolysis in neutrophils, significantly inhibiting neutrophil migration, apoptosis, and the release of ROS, myeloperoxidase (MPO), antimicrobial peptides, and IL-8 to alleviate UC (75). Glycolysis may be mediated by the PI3K/Akt-HIF-1α pathway leading to the downregulation of LDHA and thus inhibition of neutrophils. Neutrophil chemotaxis and phagocytosis are inhibited when extracellular acidification rate (ECAR) and lactate production are eliminated with the glycolytic inhibitor 2-DG (76). The specific mechanisms of neutrophil action in IBD under the glycolytic perspective are still rich to be discovered.

#### 3.2.3 Dendritic cell

Dendritic cells (DCs) are a crucial type of antigen-presenting cells that play a vital role in the immune response of the body. In addition, they are also involved in the pathogenesis of IBD, as patients with IBD have a higher number of DCs in their intestinal tissues compared to healthy individuals (77). DCs release several pro-inflammatory cytokines and express high levels of receptors that are involved in T-cell activation, such as IL-12, IL-23, and TNF-α, which induce and exacerbate intestinal inflammation (78, 79). Furthermore, DCs play a significant role in regulating the immune response of T cells by stimulating their differentiation into Th1 and Th17 cells, thereby promoting an imbalanced immune response that accelerates the progression of IBD (80, 81). On the other hand, some studies have demonstrated that DCs can induce the production of Th2, CD4+CD25+Foxp3+ regulatory T cells (Tregs), thus exerting a certain degree of immunosuppressive effect (81, 82), which can inhibit the progression of IBD. Thus, DCs play multiple roles in the pathophysiology of IBD, and their specific mechanisms of action require further investigation. DC maturation is mediated by toll-like receptors (TLR) and other pattern-recognition receptors. The use of TLR agonists stimulates a metabolic shift towards aerobic glycolysis in DCs, which involves the PI3K/AKT and AMPK pathways and is found to be inhibited by IL-10 (83).

Different DC subtypes have diverse metabolic requirements, and metabolic profiles play a critical role in their function. Similar to macrophages, mTORC1, HIF-1α, and mitochondrial fitness play important roles in DC differentiation and polarization. In the resting state, DCs rely primarily on oxidative phosphorylation and AMPK signaling to maintain their quiescent metabolic state. However, in the activated state, they turn to glycolysis to increase the production of biosynthetic precursors, thereby promoting cell growth and function (84). The rapid induction of glycolysis is identified as a component of TLR signaling, which is essential for the anabolic requirements for DC activation and function (85). Xiang et al. (86) discovered that Kinsenoside (KD) promotes PD-L1 expression via PI3K-AKT-FoxO3, reducing IL-12 secretion, inhibiting DC maturation, and blocking the activation of CD8T cells and hepatic stellate cells (HSCs), thus reducing inflammation. Lu et al. (87), also found that Smad7, a negative regulator of TGF-β signaling, restricts the PDL1/1-PD4 axis in DCs and CD2T cells to mediate intestinal inflammation. However, the role of the glycolytic component in these mechanisms has not yet been investigated, which could be considered for future studies. Targeting glycolysis to regulate DCs may be useful in the research for the treatment of inflammatory diseases, including IBD.

#### 3.2.4 T cell

As cells responsible for recognition and attack in abnormal situations such as infection, cancer, and autoimmunity, T cells have a major responsibility. T cells can be activated and differentiated into various subtypes, such as helper T cells (Th1, Th2, Th17, etc.) and regulatory T cells (Treg), which further regulate and control the extent and type of immune response (88). In IBD, T cells are considered to be one of the key pathological factors, usually associated with intestinal accumulation of pro-inflammatory Th1 and Th17 cells, accompanied by insufficient numbers of Treg and Tr1 immunosuppression (89). In the intestines of IBD patients, there are metabolic disturbances, such as enhanced glycolytic pathways and diminished oxidative phosphorylation pathways (75). This metabolic feature is also present in T cells, for example, CD8+ T cells undergo more aerobic glycolysis and glutaminolysis to achieve proliferation and effector functions (90). In T-cell biology, metabolic remodeling is intrinsically linked to cell development, activation, function, differentiation, and survival. In IBD, pro-inflammatory CD4+ T cells are not effectively regulated, leading to over-proliferation (91) and excess production of pro-inflammatory cytokines (IFN-γ, TNF-α, and IL-17), which drive inflammation and its exacerbation (92). In IBD patients, Treg cell levels are reduced (89), which can contribute to the inflammatory state. Elevating Treg cell levels can help regulate inflammation in IBD. HIF-1α is an important factor that regulates Treg cell activation (93).

Activated Treg cells need to migrate to the site of inflammatory tissues to perform their immunomodulatory functions, and this migration process is closely related to glycolysis (94). Kishore et al. (95) found that glycolysis facilitates the initiation of migration through a PI3K-mTORC2-mediated pathway, which induces glucokinase (GCK) production, and ultimately GCK binds to actin to promote cytoskeletal rearrangement for higher migratory activity. The mTOR signaling pathway has been an important bridge to studying the relationship between glycolysis and T cells. Hu et al. (96) found that a novel dual TORC1/2 inhibitor, AZD8055, promotes Treg cell differentiation in the colon of DSS-induced IBD mice, increases the percentage of Treg cells, and reduces the number of colonic CD4+ T cells, Th1 and Th17 cell activation, and cytokine production. Zhao et al. (97) also observed a similar phenomenon. This suggests that the mTOR pathway’s regulation of T cell metabolism is a promising therapeutic target in IBD.

### 3.3 Effect of glycolysis on intestinal barrier function

Gut Barrier function refers to the ability of the intestinal epithelium to act as a barrier between the external and internal environment. Its crucial role in human health has been widely recognized for a long time (98). The loss of intestinal epithelial barrier integrity, which leads to increased intestinal permeability, is a key mechanism in the pathogenesis of IBD (99). Over the years, researchers have continued to explore the mechanisms involved, including glycolysis, which plays a vital role in intestinal barrier function. SCFAs like butyrate, which are produced by the metabolism of the intestinal microbiota, have an enhancing effect on the intestinal barrier (100, 101). Glycolysis has a regulatory role in the production of SCFAs. Li et al. (47) found that treatment of DSS-induced IBD mice with barley leaves (BL) induced their intestinal microbiota to regulate metabolic reprogramming of colonic tissues and confirmed a significant enhancement of glycolytic processes by metabolic analysis. Dietary BL supplementation leads to the enrichment of the purine metabolite, inosine, in the microbiota, which activates peroxisome proliferator-activated receptor γ (PPARγ) signaling in the colonic epithelium and improves mucosal barrier function via adenosine 2A receptor (A2AR)/PPARγ. Tian et al. (102) focused their attention on the transketolase pathway linking the pentose phosphate pathway (PPP). The absence of TKT leads to significant deformation of tight junctions in colonic epithelial cells by upregulating the colonic expression of cleaved caspase-3, which interferes with glucose metabolism pathways, leading to the accumulation of PPP metabolites and the reduction of glycolytic metabolites. This reduction limits epithelial cell function by decreasing the energy supply of epithelial cells, which may be responsible for epithelial cell death. This study also suggests that although glycolysis is over-activated in IBD, its resultant effect is lower than normal, which can also lead to adverse effects. Hence, glycolysis also has an equilibrium interval, and too high or too low levels have negative consequences. Additionally, there is an interesting branch of glycolysis, the hexosamine pathway, which synthesizes uridine diphospho-N-acetylglucosamine (UDP-GlcNAc). This molecule is subsequently used for post-translational modification of proteins through glycosylation (O-GlcNAcylation) (103). A study found that protein O-GlcNAcylation levels and O-GlcNAc transferase (OGT) were reduced in intestinal epithelial cells of IBD patients. Specific OGT deficiency in intestinal epithelial cells causes epithelial barrier disruption in mice (104). However, relatively few studies have focused on this direction, the mechanisms are not yet clear, and more resources are needed.

## 4 Role of glycolysis in colorectal cancer

**Figure 3:**
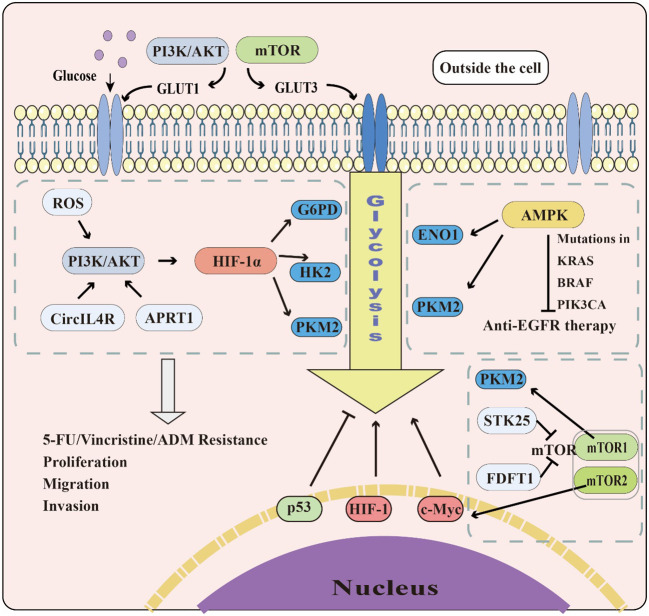
The role of glycolysis in CRC. The PI3K/AKT and mTOR pathways can promote GLUT1&3 expression to accelerate glucose transport. The PI3K/AKT, AMPK, and mTOR pathways also affect glycolysis by regulating glycolytic enzymes and transcription factors, including c-Myc, p53, and HIF-1. While c-Myc and HIF-1 promote glycolysis in cells, p53 plays the opposite role.

Colorectal cancer (CRC) is one of the leading causes of cancer incidence and mortality (12). As the incidence of CRC continues to rise in low- and middle-income countries due to Westernization (105), it is crucial for medical practitioners and researchers to investigate the mechanisms and potential therapeutic strategies for this disease. Colitis-associated colorectal cancer (CAC) is an important part of CRC. However, its related research on glycolysis is relatively less compared to general CRC, thus the need to further strengthen research in this field. One of the significant differences between cancer cells and healthy cells is metabolic reprogramming, with aerobic glycolysis promoting tumorigenesis and metastasis. The PI3K/AKT, mTOR, MAPK, Wnt, and AMPK signaling pathways play roles in regulating aerobic glycolysis in cancer cells, and transcription factors, including c-Myc, p53, and HIF-1, significantly regulate glycolysis-related enzymes. Therefore, understanding the regulation of key enzymes of glycolysis is of great value. Given the crucial role of glycolysis in CRC, a more in-depth study of its relationship with this disease, including signaling pathways, transcription factors, and glycolysis-related enzymes is crucial (**Figure 3**).

### 4.1 Signaling pathways linking glycolysis and CRC

#### 4.1.1 PI3K/AKT pathway

The PI3K/AKT pathway is a crucial regulator of cell proliferation, survival, and metabolism, and is activated by several growth factors and cytokines. Dysregulation of this pathway is often observed in cancer, making it an important event in colorectal carcinogenesis. In particular, it plays a key role in drug resistance and metastasis in CRC (32). Acquired resistance to 5-fluorouracil (5-FU) remains a major clinical challenge in the management of CRC. Analysis of glycolytic metabolic profiling has revealed increased glycolytic fluxes in 5-FU-resistant cells. Furthermore, mRNA and protein levels of the enzymes involved in glycolysis, such as hexokinase 2 (HK2), pyruvate kinase M2 (PKM2), and glucose-6-phosphate dehydrogenase (G6PD), are found to be upregulated in these cells (106). ROS accumulation, mediated through the activated PI3K/AKT signaling pathway, leads to the upregulation of HIF-1α, which promotes glycolysis (106). These findings strongly support targeting HIF-1α to modulate the PI3K/AKT signaling pathway and overcome acquired resistance to 5-FU in CRC. In addition to 5-FU resistance, the PI3K/AKT pathway has also been implicated in Vincristine Resistance (107) as well as resistance to other common chemotherapeutic agents, such as Adriamycin (ADM) and cisplatin (DDP) (108).

Moreover, the PI3K/AKT pathway has a key role in CRC metastasis. Overexpression of circRNAs (circNSUN2, circ-ERBIN, circIL4R) has been observed in CRC and shown to promote disease progression (33, 109, 110). Knockdown of circIL4R in HCT116 and DLD1 cells significantly reduced p-AKT and its downstream-related genes, such as Nanog and CyclinD1 (*CCND1*). It was found that circIL4R activates the PI3K/AKT signaling pathway in CRC cells, and the knockdown of circIL4R inhibits the proliferation, migration, and invasion abilities of CRC cells (33). In research targeting the CAC and PI3K/AKT pathway, researchers found that miR-21 activates the PI3K/AKT pathway, promotes the release of inflammatory cytokines IL-1β, IL-6, and TNF-α, and activates oncogenes during CAC development. Thus, miR-21-mediated dysregulated gene networks and chronic inflammation are behind tumorigenesis during CAC development (111). Long et al. (112) have reported that arginine ADP-ribosyltransferase1 (ART1) plays a role in regulating glycolysis in CRC. Their study confirmed that the key function of ART1 in the elevation of glucose consumption in CT26 cells is the regulation of GLUT1-dependent glycolysis in CRC through the PI3K/AKT/HIF-1α pathway. Therefore, investigating the PI3K/AKT pathway is crucial for linking glycolysis and CRC. This linkage has great value in providing a solid foundation for future targeted treatments for CRC resistance and migration.

#### 4.1.2 AMPK pathway

The AMPK pathway is a critical regulator of cellular energy homeostasis and is activated by various stress signals such as nutrient deficiency and hypoxia. Several studies have shown that abnormal mitochondrial activity, particularly dysregulation of redox and oxidative stress, is associated with CRC progression (113–115). Researchers Chen et al. (116) found that superoxide dismutase 2 (SOD2) is upregulated in CRC and the overexpression of SOD2 induces H2O2-mediated CRC by upregulating AMPK and the onset of glycolysis. Knocking down the *SOD2* gene in cells results in significant decreases in glycolytic markers, including L-lactate and MCT4. Moreover, inhibiting the AMPK pathway leads to a decrease in glycolytic activity and the migratory capacity of CRC cells. Elena et al. (117) reported that activation of the AMPK pathway mediates autophagy in CAC, but the role of autophagy in CAC is still controversial, on the one hand, it is believed that autophagy is a mechanism of tumor suppression, and on the other hand, it exists that autophagy facilitates tumor cells in a hypoxic, nutrient-poor environment. Specific studies have also found that α-enolase (ENO1), one of the glycolytic factors, affects CRC development and metastasis by regulating the AMPK pathway (118). Another glycolytic enzyme, PKM2, is similarly regulated by AMPK (119). Besides being associated with CRC development and metastasis, AMPK is also crucial in studying CRC drug resistance. Microsatellite instability, *KRAS*, *BRAF*, and *PIK3CA* gene mutations can occur in CRC and generate drug resistance, leading to a significant reduction in the effectiveness of monoclonal antibodies targeting the epidermal growth factor receptor (EGFR) in CRC treatment (120). Researchers Ye et al. (121) investigated the mechanisms of mutant KRAS-mediated resistance to anti-EGFR therapy and found that KRAS mutations inhibit AMPK phosphorylation through glycolysis. The glycolysis inhibitor 3-BrPA restores AMPK phosphorylation in cells, and the activation of AMPK inhibits the abnormal expression of myeloid cell leukemia 1 (Mcl-1), a key factor mediating drug resistance. Hence, promoting AMPK activation through influencing glycolysis can overcome KRAS-mediated anti-EGFR antibody resistance. Targeting AMPK can sensitize cancer cells to chemotherapy and radiotherapy, making this pathway a promising target for CRC treatment.

#### 4.1.3 mTOR pathway

The mammalian target of the rapamycin (mTOR) pathway is a central regulator of cell growth and metabolism, involved in a variety of cellular processes, including protein synthesis, autophagy, and energy metabolism. It integrates signals from various upstream pathways such as the PI3K/AKT pathway. mTOR is encoded by the *mTOR gene* and forms two multisubunit complexes: mTOR complex 1 (mTORC1) and mTOR complex 2 (mTORC2) (122). mTORC1 is an activator of glycolysis and can upregulate the expression of *PKM2*, *GLUT3*, and other glycolysis-related genes (123), while mTORC2 also regulates glycolysis through FoxO acetylation and upregulation of c-Myc (124). Karl et al. (125) found that in CAC, the activity of mTORC2 is decreased in human and mouse macrophages. mTORC2 controls the overactivation of proinflammatory polarization genes and inhibits the progression of CAC. Studies have shown that the serine/threonine protein kinase 25 (STK25) mitigates CRC growth by regulating glycolysis through mTOR signaling. It was found that GOLPH3 activates mTOR signaling through phosphorylation of mTORC1 and mTORC2 specific substrates, thus, STK25 affects mTOR signaling through GOLPH3 and inhibits CRC cell proliferation and glycolysis, thereby retarding the development of CRC (126). Moreover, a recent study found that fasting inhibits glycolysis as well as cell proliferation in CT26 cells and upregulates Farnesyl-Diphosphate Farnesyltransferase 1 (FDFT1), thereby inhibiting AKT/mTOR/HIF1α signaling, which then further affects glycolysis in CRC (127). Therefore, mTOR inhibition-based therapies may be a promising approach against CRC.

### 4.2 Important transcription factors linking glycolysis and CRC

#### 4.2.1 p53

p53 is a well-known tumor suppressor protein that plays an important role in regulating cell growth and division, including glycolysis (128). In terms of glucose uptake, p53 can inhibit the expression of *GLUT1, GLUT3*, and *GLUT4 genes*, thus reducing the uptake of glycolytic raw materials to achieve glycolysis inhibition (129, 130). In addition, p53 can also directly or indirectly affect the expression of glycolysis-related enzymes, such as HK2, G6PD, PFKFB3/4, PGAM1, and PHGDH (128). In a study on P53 expression levels and clinical endoscopy findings in patients with IBD as well as CAC, there was a significant negative correlation between p53 expression levels and the severity of clinical endoscopy (131). Recently, it was found that the expression of PTEN-induced kinase 1 (PINK1) was lower in the colon tissue of CRC patients than in the normal population and that disruption of PINK1 increased the probability of colon tumorigenesis in an IBD-related CRC mouse model, suggesting that PINK1 has a tumor suppressor role in CRC (132). Further studies revealed that PINK1 overexpression can induce activation of the p53 signaling pathway to promote mitochondrial autophagy and reduce glycolysis. PINK1 overexpression can significantly reduce acetyl coenzyme A production in CRC through the HIF-1α-PDHK1-PDHE1α axis, thereby inhibiting tumor growth (132). While researchers have put total effort into targeting tumors with mutant p53, which is mutated up to 50% of the time in CRC, the relative lack of studying CRC with wild-type p53 is noteworthy. A recent study showed that METTL14 can be transcriptionally activated by wild-type p53 and can inhibit the expression of SLC2A3 and PGAM1, thus suppressing aerobic glycolysis, CRC malignant phenotype of p53-WT CRC (133). As an upstream regulator of the Warburg effect, p53 has also been shown to be of great potential value in determining CRC prognosis (134).

#### 4.2.2 HIF-1

HIF is a transcription factor that regulates cellular responses to hypoxic levels. HIF-1 is a heterodimer composed of the constitutively expressed subunit HIF-1β and the oxygen-regulated subunit HIF-1α (135). Among these, HIF-1α plays a crucial role in glycolysis-related studies. HIF-1α is a key regulator of the Warburg effect, promoting glycolysis and lactate production by inducing the expression of genes related to glucose uptake (GLUT1&4, etc.) and glycolytic enzymes (LDH, etc.) (136). Zhang et al. (137) found that the intestinal fungus *Candida tropicalis (C. tropicalis*) can enhance the immunosuppressive function of myeloid-derived suppressor cells (MDSC) to promote the development of CRC. *C. tropicalis* stimulation significantly enhanced glycolysis by increasing the level of glucose uptake by MDSCs, along with the production of more lactate and the appearance of elevated levels of extracellular acidification rate (ECAR). The immunosuppressive capacity of MDSCs was effectively attenuated by treatment with the glycolysis inhibitor 2DG, demonstrating that glycolysis mediates the effect of MDSCs. It was further found that *C. tropicalis* treatment of MDSCs increased the interaction between the glycolytic enzyme PKM2 and HIF-1α, which increased the stability of HIF-1α and thus glycolysis (137). Wei (138) et al. reported that lactate, a product of glycolysis, inactivates proline hydroxylase (PHD), thereby stabilizing HIF-1α in THP-1 monocytes and subsequently promoting glycolysis and CAC growth. Moreover, recent studies have found that TNF receptor-associated protein 1 (TRAP1) is also involved in regulating hypoxia-induced HIF-1α stabilization and glycolytic metabolism in CRC species. Silencing TRAP1 in HCT116 cells led to a decrease in GLUT1 expression, reduced lactate production, and at the transcriptional level, suppression of HIF-1α-driven reprogramming of gene expression in cancer cells, indicating that TRAP1 is a possible key factor in maintaining HIF-1α-induced gene/metabolic reprogramming under hypoxic conditions (139). Although relatively few studies have investigated the relationship between iron death and HIF-1α in CRC, recent studies have found that HIF-1α plays an essential role in exacerbating diabetic nephropathy and tubular damage (140), indicating a promising direction for further investigation. HIF-1 has been intensively studied for many years, and research on HIF-1 is still persistently advancing due to its irreplaceable and significant role in thoroughly mapping its regulatory mechanisms.

#### 4.2.3 c-Myc

c-Myc is a proto-oncogene that regulates cell proliferation, differentiation, and apoptosis. Dysregulation of c-Myc has been associated with the development of various cancers (141), including CRC. In CRC, c-Myc is overexpressed and associated with tumor aggressiveness, metastasis, and poor prognosis (142). Several studies have investigated the mechanisms of c-Myc involvement in CRC pathogenesis. Yuan et al. (143) found that protein expression of glycolysis-related genes such as *HK2, PKM2, GLUT1*, and *LDHA*, is severely suppressed after treatment with the c-Myc inhibitor 10058-F4 in RKO and CaCO-2 cells. c-Myc ubiquitination causes targeted proteasome-mediated degradation (144). Tang et al. (145), on the other hand, found that the CRC glycolysis-related long non-coding RNA (GLCC1) could upregulate the protein levels of c-Myc and the interaction between c-Myc and HSP90. By detecting the level of the c-Myc ubiquitination marker (PT58), it was found that GLCC1 downregulates the ubiquitination level of c-Myc and enhances its stability, thus further promoting the development of CRC caused by glycolysis. In the study of CAC, it was found that polysaccharides of A. bracteate (ABP) treatment could activate Signal Transducer And Activator Of Transcription 3 (STAT3) in the colonic tissue of CAC mice, which further inhibited c-Myc expression, decreasing the inflammatory response inhibiting the progression of CAC (146). Despite significant progress in understanding the role of c-Myc in CRC, effective treatment against c-Myc remains challenging due to its complex regulatory network and important role in normal cell physiology. However, novel approaches, such as RNA interference, small molecule inhibitors, and gene editing technologies, are currently being investigated as potential therapeutic strategies for targeting c-Myc in CRC. In conclusion, c-Myc is a promising therapeutic target for CRC, and further research is needed to fully understand its role in disease pathogenesis and to develop effective therapeutic approaches.

### 4.3 Glycolysis-related enzymes and CRC

CRC development involves various genetic and molecular alterations, including dysregulation of glycolysis-related enzymes. Glycolysis converts glucose to pyruvate and plays a key role in energy metabolism, biosynthesis, and redox homeostasis in cancer cells. Numerous studies have explored the relevance of glycolysis-related enzymes to the pathogenesis of CRC, identifying a range of substances involved in its regulation (**Table 2**). For example, melatonin combined with hyperbaric oxygen therapy (HBO) inhibits the degree of AKT activation induced by oncogenic mutations in CRC, subsequently decreasing the expression of HIF-1 and downregulating the expression of glycolysis-related enzymes (HK2/PFK1/PKM2/LDH), limiting tumor development (147). Circular RNA hsa_circ_0005963 (ciRS-122) promotes glycolysis and CRC chemoresistance by upregulating miR-122, which leads to PKM2 upregulation (148). Parthenolide derivatives reduce PKM2 expression in CRC cells, inhibit the PKM2/STAT3 signaling pathway, and PKM2 dimerization in nuclear translocation, inhibiting CRC tumor growth (149). Betulinic acid (BA) inhibits the glycolytic enzymes (HK2/FK1/PEP/PKM2) in CRC cells (150). Moreover, Atractylenolide I downregulates HK2 expression and blocks the JAK2/STAT3 pathway in CRC cells, inducing cellular regulation and alleviating CRC (151). Oxymatrine also inhibits PKM2 activity and expression in CRC (152). Natural shikonin (SK) and acetyl-shikonin (acetyl-SK) modulate the disorganized intestinal microbiota in CAC mice and restore the abnormally up-regulated PKM2 and pro-inflammatory cytokines (IL-1β,IL-6 and TNF-α) (153). In another study, MiR-103a-3p upregulated PKM1/LDHA/PFK1/HK2 activity, facilitating CRC cell invasion and metastasis (154). Non-SMC Condensin II Complex Subunit D3 (NCAPD3) is overexpressed in CRC, interacts with c-Myc, upregulating GLUT1, HK2, ENO1, PKM2, and LDHA gene expression, ultimately enhancing cellular aerobic glycolysis (155). From these studies, the importance of PKM2 and HK2 in glycolysis and their regulatory mechanisms is relatively clear. However, the role of other glycolysis-related enzymes should not be ignored and explored appropriately.

**Table 2:** Glycolysis-related enzymes and their regulators.

Substance	Targeted enzyme	Glycolysis variation trend	Key effects	References
Melatonin	HK2/PFK1/PKM2/LDH	**↓**	Restrains cancer stemness pathway, inflammation, and immune escape	(147)
CiRS-122	PKM2	**↑**	Reverses resistance to oxaliplatin	(148)
Parthenolide derivatives	PKM2	**↓**	Suppresses tumor growth	(149)
Betulinic acid	HK2/PFK1/PEP/PKM2	**↓**	Suppresses proliferation and glucose uptake	(150)
Atractylenolide I	HK2	**↓**	Induces apoptosis	(151)
Oxymatrine	PKM2	**↓**	Inhibits cancer cell migration and invasion	(152)
SK & acetyl-SK	PKM2	**↓**	Improves species richness	(153)
MiR-103a-3p	PKM1/LDHA/PFK1/HK2	**↑**	Promotes HIF-1α expression	(154)
NCAPD3	HK2/ENO1/PKM2/LDHA	**↑**	Inhibits PDH activity and TCA cycle	(155)

## 5 Conclusion and perspective

Glycolysis is a metabolic process that plays a crucial role in a variety of diseases, including metabolic, neurological, vascular, inflammatory, and oncological diseases. There is sufficient evidence that glycolysis influences intestinal microbiota, the immune system, and intestinal barrier function in IBD, and is involved in signaling pathways and transcription factors in CRC pathogenesis. Understanding the role of glycolysis in disease pathogenesis may provide new therapeutic opportunities for the treatment of IBD and CRC. Targeting glycolysis-related enzymes and signaling pathways may be an effective way to develop new therapies for these diseases. Additionally, understanding the relationship between glycolysis and the immune system may lead to new immunomodulatory treatments for IBD and CRC. The development of glycolysis inhibitors as potential therapeutic agents is ongoing and is an active area of research. Future research should focus on developing more potent and selective inhibitors targeting glycolysis-related enzymes and signaling pathways. It is also important to investigate the use of glycolytic inhibitors in combination with other therapies to enhance their therapeutic potential. In conclusion, this paper provides valuable insights into the role of glycolysis in IBD and CRC and highlights the need for further studies to fully understand the mechanisms involved in developing effective therapeutic approaches targeting glycolysis.

## Author contributions

Material preparation and analysis were performed by YX, LZ, DO, QT, FM, and XS. The first draft of the manuscript was written by YX, DO, LZ, and QT. The manuscript was proof-read and edited by XS and FM, and all authors commented on previous versions of the manuscript. All authors contributed to the article and approved the submitted version.
